# Prediction of Refractive Error Based on Ultrawide Field Images With Deep Learning Models in Myopia Patients

**DOI:** 10.3389/fmed.2022.834281

**Published:** 2022-03-30

**Authors:** Danjuan Yang, Meiyan Li, Weizhen Li, Yunzhe Wang, Lingling Niu, Yang Shen, Xiaoyu Zhang, Bo Fu, Xingtao Zhou

**Affiliations:** ^1^Eye Institute and Department of Ophthalmology, Eye & ENT Hospital, Fudan University, Shanghai, China; ^2^NHC Key Laboratory of Myopia, Fudan University, Shanghai, China; ^3^Key Laboratory of Myopia, Chinese Academy of Medical Sciences, Shanghai, China; ^4^Shanghai Research Center of Ophthalmology and Optometry, Shanghai, China; ^5^Shanghai Engineering Research Center of Laser and Autostereoscopic 3D for Vision Care, Shanghai, China; ^6^School of Data Science, Fudan University, Shanghai, China; ^7^Shanghai Medical College, Fudan University, Shanghai, China

**Keywords:** refractive error prediction, myopia, deep learning, ultrawide field imaging, ResNet-50, Inception-V3, Inception-ResNet-v2

## Abstract

**Summary:**

Ultrawide field fundus images could be applied in deep learning models to predict the refractive error of myopic patients. The predicted error was related to the older age and greater spherical power.

**Purpose:**

To explore the possibility of predicting the refractive error of myopic patients by applying deep learning models trained with ultrawide field (UWF) images.

**Methods:**

UWF fundus images were collected from left eyes of 987 myopia patients of Eye and ENT Hospital, Fudan University between November 2015 and January 2019. The fundus images were all captured with Optomap Daytona, a 200° UWF imaging device. Three deep learning models (ResNet-50, Inception-v3, Inception-ResNet-v2) were trained with the UWF images for predicting refractive error. 133 UWF fundus images were also collected after January 2021 as an the external validation data set. The predicted refractive error was compared with the “true value” measured by subjective refraction. Mean absolute error (MAE), mean absolute percentage error (MAPE) and coefficient (*R*^2^) value were calculated in the test set. The Spearman rank correlation test was applied for univariate analysis and multivariate linear regression analysis on variables affecting MAE. The weighted heat map was generated by averaging the predicted weight of each pixel.

**Results:**

ResNet-50, Inception-v3 and Inception-ResNet-v2 models were trained with the UWF images for refractive error prediction with *R*^2^ of 0.9562, 0.9555, 0.9563 and MAE of 1.72(95%CI: 1.62–1.82), 1.75(95%CI: 1.65–1.86) and 1.76(95%CI: 1.66–1.86), respectively. 29.95%, 31.47% and 29.44% of the test set were within the predictive error of 0.75D in the three models. 64.97%, 64.97%, and 64.47% was within 2.00D predictive error. The predicted MAE was related to older age (*P* < 0.01) and greater spherical power(*P* < 0.01). The optic papilla and macular region had significant predictive power in the weighted heat map.

**Conclusions:**

It was feasible to predict refractive error in myopic patients with deep learning models trained by UWF images with the accuracy to be improved.

## Introduction

Myopia is one of the most common causes of distance vision impairment with its global incidence continuing to increase each year (1, 2). High myopia and its related retinopathies have been reported to be one of the most common causes of blindness (3). Tessellated fundus, lacquer cracks, focal or diffuse chorioretinal atrophy are all typical fundus findings of myopia, especially high myopia, in clinical settings (4). Deep learning models have already been successfully trained by fundus images in assessing glaucoma (5), diabetic retinopathy (6), age-related macular degeneration (7, 8) and retinopathy of prematurity (9). With the help of ultrawide field (UWF) imaging, the visualized fundus photograph obtained an unprecedented large-angle view of up to 200° and achieved a wide application in ophthalmic settings (10–13).

Optomap UWF images were gained from a green (532 nm) and a red (633 nm) laser wavelength scanning and were superimposed from the red and green channels by the software to yield semirealistic color, different from the true color of the traditional fundus photographs. While UWF imaging was originally utilized to assess the fundus pathologies, Varadarajan et al. explored the feasibility of training a deep learning model for predicting the refractive error *via* traditional fundus images (14). However, such feasibility has not been verified on UWF images. It remained unknown whether the peripheral retinal area, unavoidable eyelids or eyelashes would produce noise for the prediction or not. Up to now, fundus images utilized for deep learning training were mainly traditional fundus images with a 30 or 45° view (15, 16).

It is worth exploring the potential of predicting the refractive error *via* UWF images. The purpose of this study is to explore the feasibility of applying UWF images for deep learning training to predict the refractive error of myopic patients.

## Methods

### Datasets and Image Acquisition

Nine hundred and eighty seven fundus images of 987 patients' left eyes were photographed by Optomap Daytona scanning laser ophthalmoscope (Daytona, Optos, UK) under dual lasers at 532nm and 633nm to gain the 200° pseudo-color fundus images. The images were obtained from November 2015 to January 2019 in Eye and ENT Hospital of Fudan University. Only patients with myopia in both eyes were included in this study. 133 UWF fundus images were collected after January 2021 as the external validation set to test the performance ResNet-50, Inception-v3 and Inception-ResNet-v2 deep learning of the three models. Only left eyes were chosen since data obtained from the both eyes of the same patient were regarded correlated. All the enrolled patients were myopia patients seeking for refractive surgery treatment. Patients with ocular diseases besides myopia (e.g., diseases that affected fundus imaging like cataract or vitreoretinal diseases or glaucoma), history of trauma or any ocular surgery were all excluded. All enrolled images were gradable with the fovea located in the center. The images were regarded as gradable when there was no blurring of the optic disc or foveal area and less than 50% peripheral retinal area covered by eyelids or eyelashes.

**Figure 1:**
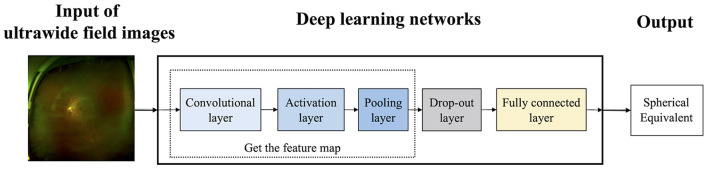
The training process of deep learning models.

UWF images were exported in JEPG forms and compressed to 512 ^*^ 512 pixels for analysis. The training processes of deep learning models by UWF images for spherical equivalent prediction were summarized in Figure 1. This study complied with the requirements of the Ethics Committee of Eye and ENT Hospital of Fudan University (No. 2020107) and was conducted following the principles of the Declaration of Helsinki.

### Network Structure

The deep learning neural network models applied pixel values of UWF fundus images in a series of mathematical calculations, which was the process of the models “learning” how to calculate the spherical equivalent. During the training process, the parameters of the neural network were initially set to random values. For each image in the training set, the predictive values given by the model were compared with the known labels, namely spherical equivalent in this study. Refractive parameters were measured by an experienced optometrist through subjective refraction with the phoropter (NIDEK RT-5100, Japan). Spherical equivalence was used as the label for refractive prediction. Spherical equivalence (SE) equaled spherical power (D) plus 1/2 ^*^ cylindrical power (D). With proper adjustments and sufficient data, the deep learning model could predict the refractive error on the new image.

Three deep learning models (ResNet-50, Inception-v3 and Inception-ResNet-v2 models) were trained by UWF images for refractive error prediction. 987 fundus images were divided into the training data set (790 UWF images) and the internal test set (197 UWF images) with a ratio of 8:2.

The deep learning models were all built following the Apache2.0 license and written in Python 3.6.6. TensorFlow-GPU 1.12.0 was used as the backend. Keras was adopted as the neural network application programming interface. Keras (https://keras.io) is an open-source artificial neural network library written in Python that serves as an application interface to TensorFlow. Keras supports many artificial intelligence algorithms and serves as a platform for building deep learning models of designing, debugging, evaluation and application.

To visualize the weights of the predicted power of each part of the UWF images, the image features were used as input and the weights predicted by each pixel were averaged to generate the heat map that represented the weight of predicted refractive power.

### Algorithm Evaluation

Mean absolute error (MAE), mean absolute percentage error (MAPE) and coefficient value (*R*^2^) of refractive prediction were calculated in the test set to assess the predictive performance. MAE (Mean Absolute Error) is defined as the average of the absolute difference between the predicted value and the true value as follows. MAPE (Mean Absolute Percentage Error) is another measure of prediction accuracy defined by the following formula.

The sample size is *n*, the true value of each sample is *y*_*i*_, and the predicted value is $\hat{y_{i}}$.


$$MAE=\frac{1}{n}\sum_{i=1}^{n}\left|{\hat{y}}_{i}-y_{i}\right|$$

$$MAPE=\frac{100\%}{n}\sum_{i=1}^{n}\left|\frac{{\hat{y}}_{i}-y_{i}}{y_{i}}\right|$$

### Statistical Analysis

Kruskal-Wallis rank sum test was used to compare the characteristics of participants between different datasets. One-way ANOVA was applied to compare the differences in both MAE and MAPE between different refractive error groups. Tukey multiple comparison test was adopted to assess the differences between each two groups. The Spearman rank correlation test was used to perform univariate analysis and multivariate linear regression analysis on variables that affected the MAE value. Statistical analyses were performed with SPSS version 22.0 (IBM Corp, New York). *P* < 0.05 was considered statistically significant.

## Results

### Characteristics of the Participants

Nine hundred and eighty seven enrolled patients (male/female: 274/713) were aged 27.68 ± 7.04 years (range: 16 - 55 years). The averaged spherical equivalent was−11.17±-4.41D (range: −1.25-−28.88D). Spherical power averaged −10.53±-4.64D and cylindrical power averaged −1.73 ± 1.27D. The axial length averaged 27.85 ± 1.99mm, ranging from 20.67mm to 37.15mm.

One hundred and thirty three patients (male/female: 23/110) enrolled for the external validation were aged 27.76 ± 5.53 years. The averaged spherical equivalent was−9.03 ± 2.79D. The axial length averaged 27.19 ± 1.50mm. The distributions of refractive error of enrolled eyes in the training set, the test set, the whole data set and the external validation set were shown in Supplementary Figure 1. The patient characteristics of the training set, the test set, the whole data set and the external validation set were detailed in Supplementary Table 1.

### Performance of Deep Learning Models in Refractive Prediction

The ResNet-50, Inception-v3 and Inception-ResNet-v2 models trained by UWF images for predicting spherical equivalent were with R^2^ of 0.9562, 0.9555, 0.9563 in the test set. MAE of the three deep learning models was 1.72D (95%CI: 1.62–1.82D), 1.75D (95%CI: 1.65–1.86D) and 1.76D (95%CI: 1.66–1.86D), respectively. MAE was of no statistical difference in the three deep learning models. MAPE of the above three deep learning models was 61.01% (95%CI: 54.19–67.82%), 40.50% (95%CI: 33.64–47.35%) and 36.79% (95%CI: 30.05–43.52%).

**Figure 2:**
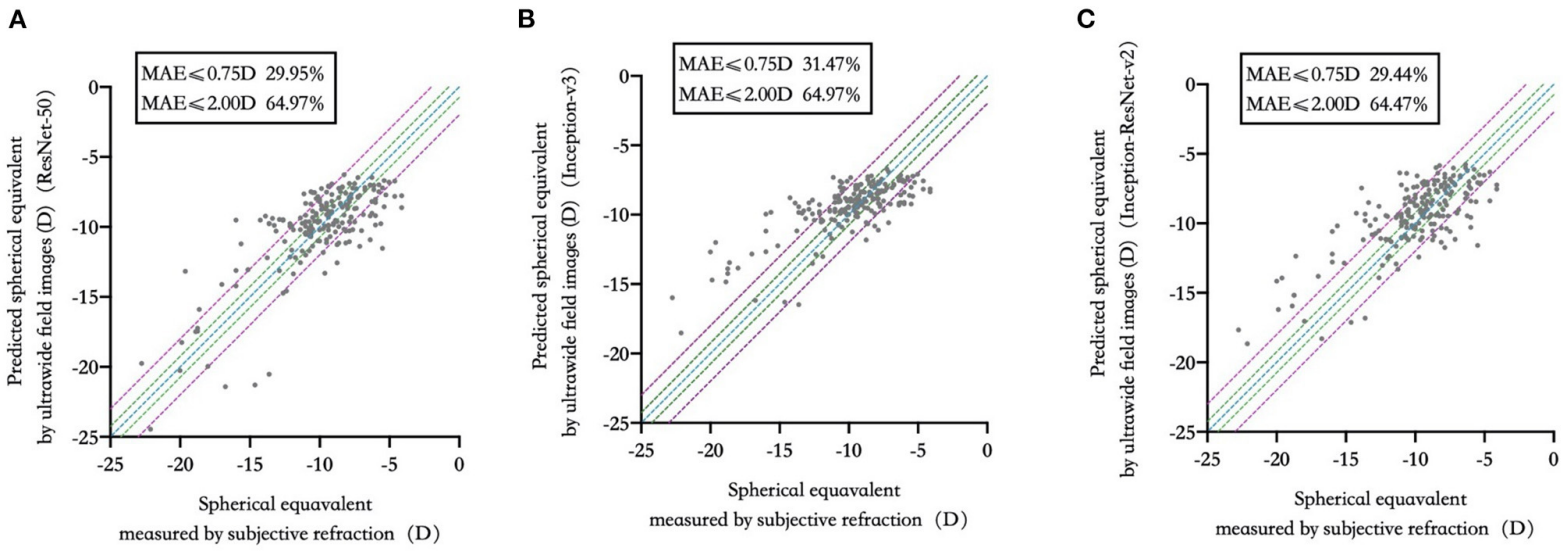
Predicted spherical equivalent versus that measured by subjective refraction in three deep learning models. **(A)** ResNet-50; **(B)** Inception-v3; **(C)** Inception-ResNet-v2.

29.95, 31.47, and 29.44% of the test set were within 0.75D of deviation from the “true value” measured by subjective refraction. 64.97, 64.97, and 64.47% of the test set were within 2.00D of deviation from “true value”. The comparison of predicted spherical equivalent in the test set and that measured by subjective refraction was shown in Figure 2. Detailed distribution of MAE was summarized in Supplementary Figure 2.

### Performance of Deep Learning Models in the External Validation

MAE of the external validation was 1.94D (95%CI: 1.63–2.24D), 1.79D (95%CI: 1.53–2.06D) and 2.19D (95%CI: 1.91–2.48D) in the trained ResNet-50, Inception-v3 and Inception-ResNet-v2 models, respectively. MAE was of no statistical difference in the external validation of the three deep learning models. *R*^2^ was 0.9265, 0.9148, 0.9330 in the three deep learning models in the external validation. 27.07, 27.07, and 21.80% of the external validation set were within 0.75D of deviation from the “true value” measured by subjective refraction. 64.66, 64.66, and 53.38% of the external validation set were within 2.00D of deviation from “true value”. The comparison of predicted spherical equivalent in the external validation set and that measured by subjective refraction was shown in Supplementary Figure 3.

### MAE and MAPE of Different Refractive Error Groups

**Figure 3:**
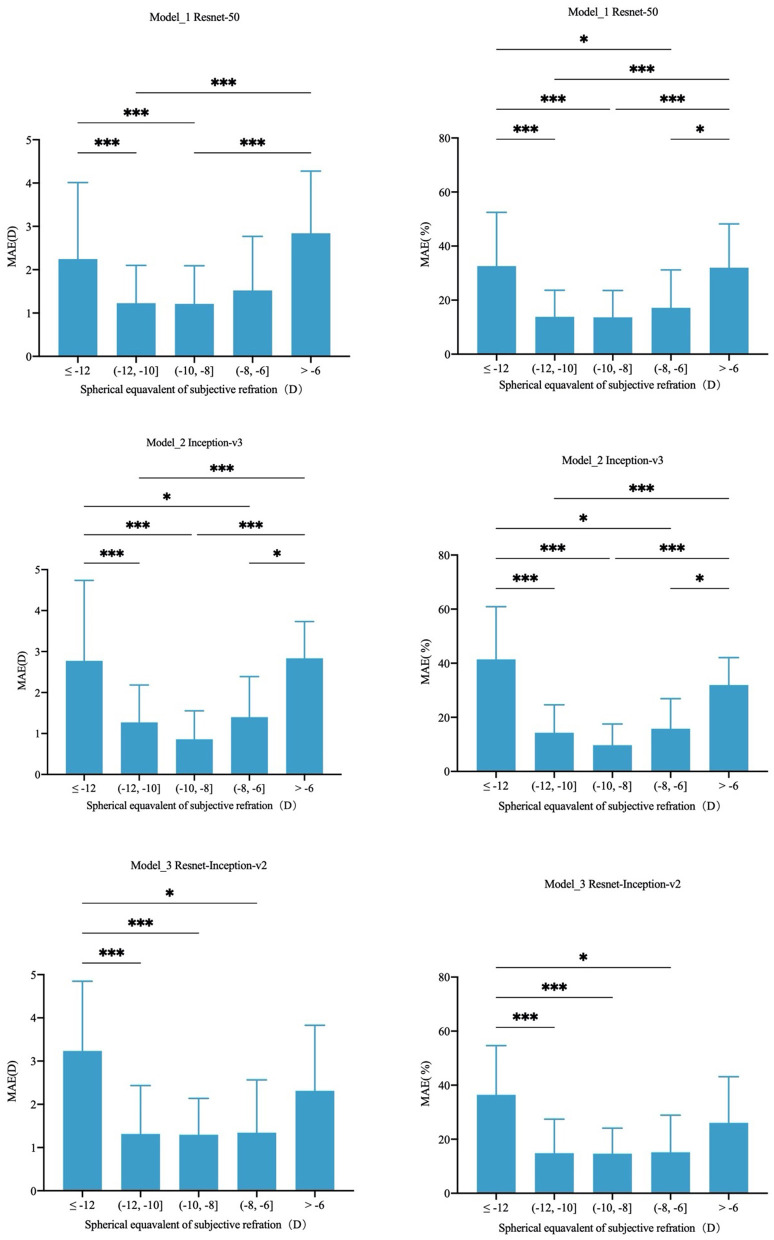
Comparison of MAE and MAPE in different refractive error groups. *P < 0.05, ***P < 0.001.

−10.00D to −8.00D group, −12.00D to −10.00D group and −8.00D to −6.00D group shared the least MAE with no significant difference (*P* > 0.05) in all three models. The least MAE was 1.29D (95%CI: 1.07–1.52D),1.21D (95%CI: 0.98–1.44D) and 0.86D (95%CI: 0.67–1.04D) in the Inception-ResNet-v2, ResNet-50 and Inception-v3 model, respectively. MAPE was the least in −10.00D to −8.00D group and −12.00D to −10.00D group with no statistical difference (*P* > 0.05) in the three deep learning models. The least MAPE was 12.29% (95%CI: 9.23–15.34%), 11.46% (95%CI: 9.06–13.86%), and 9.57% (95%CI: 7.50–11.64%) in the Inception-ResNet-v2, ResNet-50 and Inception-v3 model, respectively. Detailed comparisons between different groups were shown in Figure 3.

### Analysis of Factors Affecting the MAE of Refractive Error Prediction

**Figure 4:**
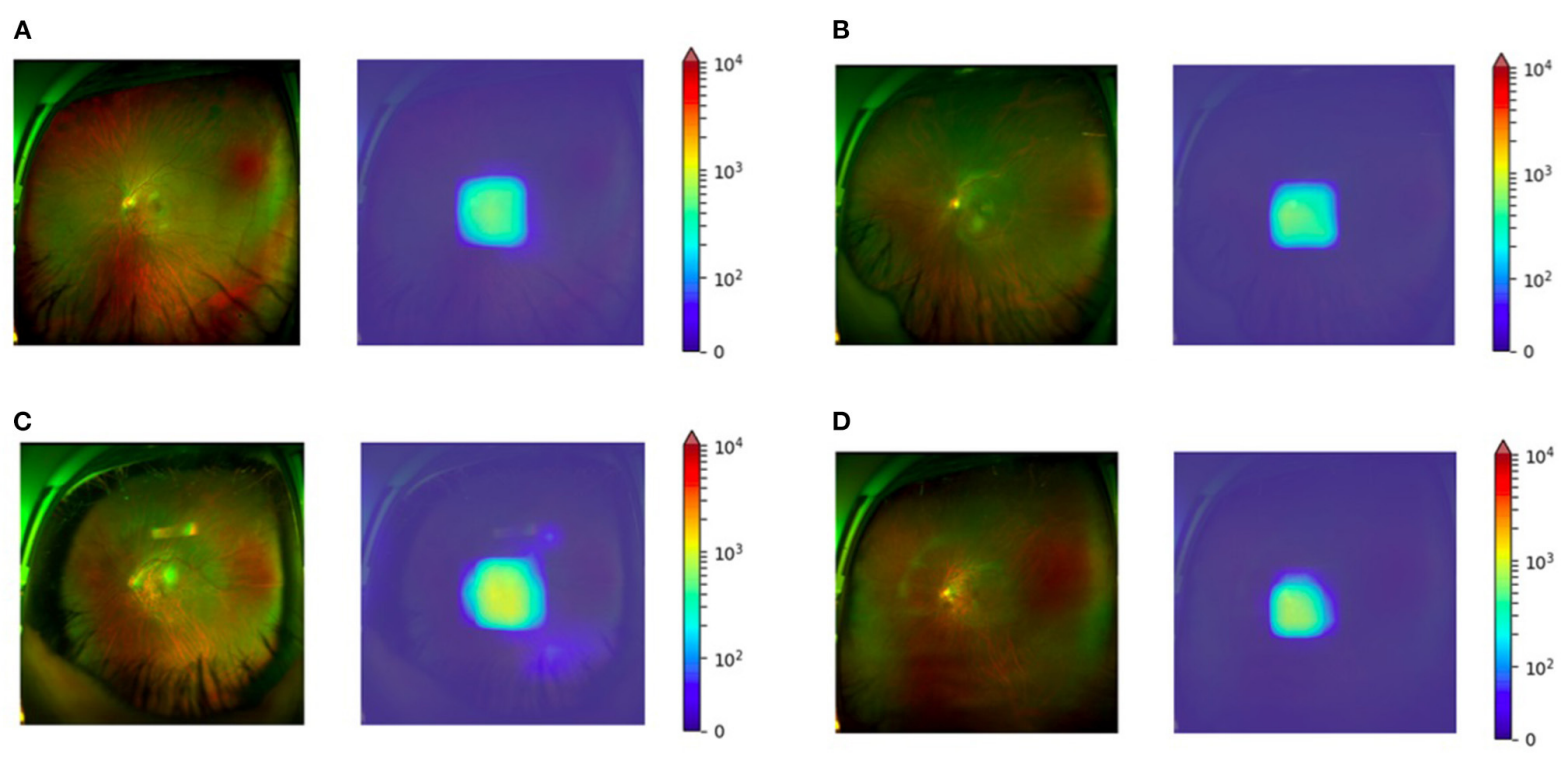
Weighted heat map for four myopic eyes SE between −6.00D to −12.00D. **(A)** Subjective refraction of −8.50D with the predicted value of −8.37D. **(B)** Subjective refraction of −9.13D with the predicted value of −8.89D. **(C)** Subjective refraction of −10.63D with the predicted value of −10.52D. **(D)** Subjective refraction of −12.00D with the predicted value of −11.65D.

The MAE was related to the older age (*P* < 0.01) and the greater spherical power (*P* < 0.01). Univariate and multivariate analyses of parameters associated with MAE were detailed in Table 1. Examples of typical UWF images in the test set with MAE ≤ 0.5D were shown in Figures 4A–D. The macular and optic papilla area of UWF images made a significant contribution to refractive error prediction in the weighted heat map.

**Table 1:** Parameters influencing MAE in deep learning models.

**Univariate analysis**	**Model_1**		**Model_2**		**Model_3**		**Multivariate analysis**	**Model_1**		**Model_2**		**Model_3**	
**Parameters**	***P***	**Correlation coefficient**	***P***	**Correlation coefficient**	***P***	**Correlation coefficient**	**Parameters**	***P***	**Correlation coefficient**	**P**	**Correlation coefficient**	**P**	**Correlation coefficient**
Age	0.006^*^	0.195	0.000^*^	0.249	0.001^*^	0.230	Age	0.010^*^	0.185	0.001^*^	0.204	0.003^*^	0.197
Sex	0.986	NS	0.846	NS	0.501	NS	SE(D)	0.023	NS	0.765	NS	0.511	NS
SE (D)	0.080	NS	0.001^*^	−0.243	0.002^*^	−0.219	Sphere (D)	0.014^*^	−0.175	0.000^*^	−0.300	0.000^*^	−0.365
Sphere (D)	0.058	NS	0.000^*^	−0.246	0.001^*^	−0.244	LOGMAR BCVA	0.561	NS	0.010^*^	0.217	0.405	NS
Cylinder (D)	0.477	NS	0.832	NS	0.541	NS	Axial length (mm)	0.401	NS	0.683	NS	0.257	NS
LOGMAR BCVA	0.008^*^	0.189	0.000^*^	0.257	0.001^*^	0.226	K1		NA	0.453	NS		NA
Axial length (mm)	0.055	NS	0.015^*^	0.173	0.018^*^	0.169	K2		NA	0.313	NS		NA
Intraocular pressure (mmHg)	0.573	NS	0.837	NS	0.980	NS							
K1	0.956	NS	0.023^*^	0.162	0.238	NS							
K2	0.859	NS	0.021^*^	0.164	0.456	NS							

* *When p is significative*.

## Discussion

Though deep learning models trained by fundus imaging have constantly been utilized for the detection and grading of ophthalmic pathologies (15, 17, 18), few studies have applied fundus photographs for refractive error prediction (14, 19).

Deep learning models trained by traditional 45 and 30° fundus photographs from the UK Biobank and the Age-Related Eye Disease Research Database could reach the MAE of 0.56D (95% CI: 0.55–0.56 D) and 0.91D (95% CI: 0.89–0.93 D) for refractive prediction (14) while most of the patients were low-grade myope or hyperope. Compared with refractive prediction utilizing 7,307 UWFI fundus images, the MAE could reach 1.115 D with more than a half of enrolled eyes were moderate myopia(−6D ≤ SE < −3D) (19).

It is worth noticing that the enrolled patients in this study were with much higher myopia than those of the previous studies. More than 90% of the patients in this study were more than −6.00D. Thus, the concept of MAPE was introduced in this study. MAPE is of vital importance in clinical practice. For example, MAE of 2.00 D indicated a huge deviation if a patient's “true” SE was −0.50D, while the same 2.00D predictive error was a minor and insignificant error for a patient with SE of −12.00D.

From the perspective of MAPE, the predictive error *via* UWF images in this study was relatively smaller than that of the previous study utilizing traditional fundus images and comparable to deep learning models trained by 7,307 UWFI fundus images in certain refractive groups. The deep learning models trained in this study were capable of refractive prediction from UWF images, but the obtained MAE so far was not enough for directly guiding the prescription of eyeglasses or as a reference before refractive surgery.

The MAPE of different myopia groups showed that MAPE increased in SE ≤ −12.00D and low-to-moderate myopia (SE > −6.00D) groups, which was attributed to the imbalanced refractive distribution of enrolled samples.

Older age was found to be related to greater MAE in all three deep learning models. It could be attributed to the darkening of the foveal reflection because of aging (20, 21). The MAE was also found to be related to spherical power and had barely relation with cylindrical power, which was consistent with clinical experience. It is the excessive elongation of the globe that plays an important role in the development of myopia and certain fundus degenerative changes like posterior staphyloma, lacquer cracks (22). Astigmatism is the result of irregularity of the cornea or lens, the information of axial length, choroid thickness and retinal portraits was rarely “stored” in astigmatism.

The weighted heat map showed that the macular and optic papilla area contributed the most in predicting the refractive error, which is also consistent with the clinical experience. Myopia, especially high myopia, could result in the thinning of the choroid layer at the macula (23). Although the size of the optic papilla has proven to be unrelated to the degree of myopia (24, 25), myopia still affects the morphology of the optic disc, e.g., optic disc tilt, rotation, torsion and the angle between the superior temporal and inferior temporal arteries of the retina (26). Another reason might be that the brightness of the optic papilla area exceeds the rest area in the UWF imaging dual-color channel.

The limitations of this study were, firstly, the sample size was relatively small. The sample size required for the deep model of training may better reach tens of thousands. Secondly, only data augmentation and dropout layer were applied to prevent over fitting in deep learning training without further separating the validation dataset. Thirdly, part of examined eyes could be minimally too close to or too far from the optimal capturing distance, causing the overall image color to be reddish or greenish.

## Conclusion

Ultrawide field fundus images could be applied in deep learning training to predict the refractive error of myopic patients with the accuracy to be improved.

## Data Availability Statement

The raw data supporting the conclusions of this article will be made available by the authors, without undue reservation.

## Author Contributions

DY: conceptualization, data collection, manuscript drafting, critical revision, and statistical analysis. ML: conceptualization, data collection, manuscript drafting, and critical revision. WL: conceptualization, data collection, and manuscript drafting. YW: manuscript drafting. LN, YS, and XZha: data collection. BF: conceptualization, critical revision of manuscript, funding, management, and supervision. XZho: conceptualization, critical revision of manuscript, funding, management, and supervision. All authors approved the final submission of this manuscript.

## Funding

This work was supported by National Natural Science Foundation of China (Grant No. 81770955), Joint research project of new frontier technology in municipal hospitals (SHDC12018103), Project of Shanghai Science and Technology (Grant No. 20410710100), Clinical Research Plan of SHDC (SHDC2020CR1043B), Project of Shanghai Xuhui District Science and Technology (2020-015), Shanghai Rising-Star Program (21QA1401500), China National Natural Science Foundation (Grant No. 71991471 and Grant No. 12071089) and the 5th Three-year Action Program of Shanghai Municipality for Strengthening the Construction of Public Health System (GWV-10.1-XK05).

## Conflict of Interest

The authors declare that the research was conducted in the absence of any commercial or financial relationships that could be construed as a potential conflict of interest.

## Publisher's Note

All claims expressed in this article are solely those of the authors and do not necessarily represent those of their affiliated organizations, or those of the publisher, the editors and the reviewers. Any product that may be evaluated in this article, or claim that may be made by its manufacturer, is not guaranteed or endorsed by the publisher.

## Supplementary Material

Supplementary Figure 1 The distributions of refractive error of enrolled eyes. **(A)** training set; **(B)** test set; **(C)** whole data set; **(D)** External validation set.

Supplementary Figure 2 Distribution of MAE of predicted spherical equivalent. **(A)** ResNet-50; **(B)** Inception-v3; **(C)** Inception-ResNet-v2.

Supplementary Figure 3 Distribution of MAE of predicted spherical equivalent in the external validation set. **(A)** ResNet-50; **(B)** Inception-v3; **(C)** Inception-ResNet-v2.

Supplementary Table 1 Patient characteristics of the training set, test set, whole data set and the external validation set.

## Abbreviations

UWFI: ultrawide field imaging
CI: confidence interval
D: diopter.
