# Supplementary figures and images for: Prediction of Refractive Error Based on Ultrawide Field Images With Deep Learning Models in Myopia Patients
Source: Front Med (Lausanne). 2022 Mar 30;9:834281. doi: 10.3389/fmed.2022.834281 (PMC9007166; doi:10.3389/fmed.2022.834281)

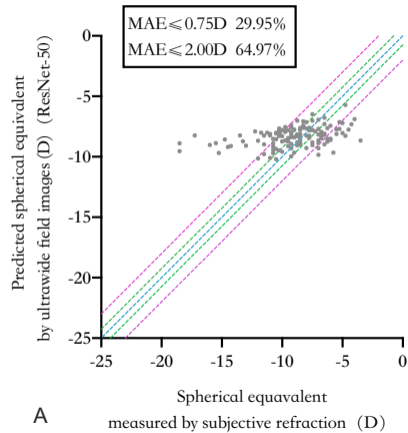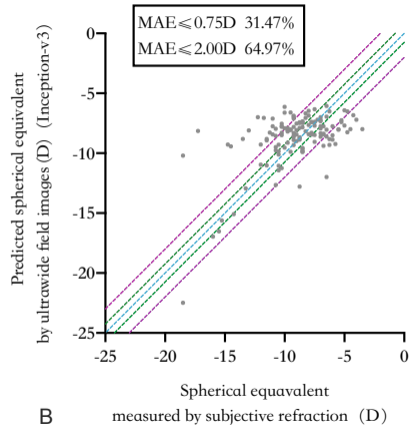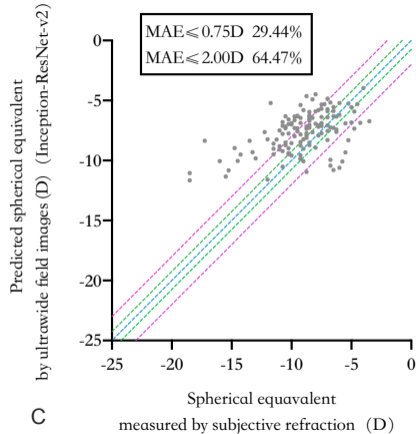

Supplement: Supplementary Figure 1 — The distributions of refractive error of enrolled eyes. (A) training set; (B) test set; (C) whole data set; (D) External validation set. [file Image_1.pdf]

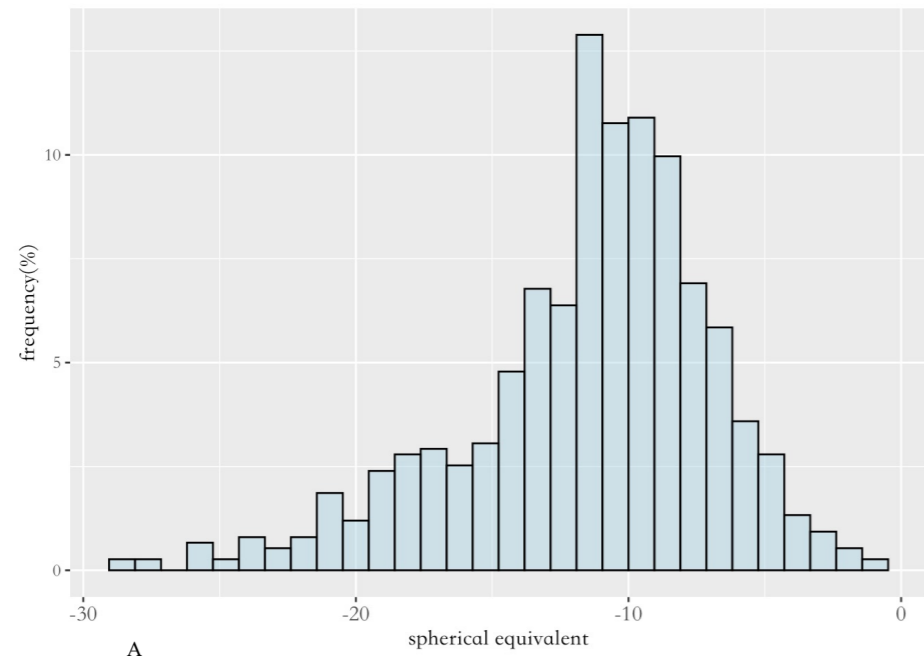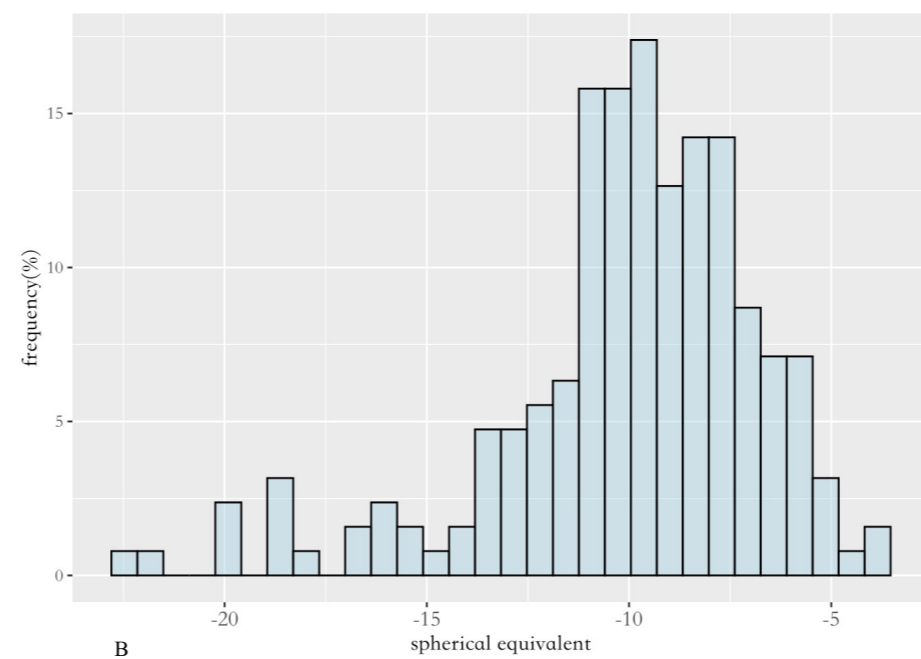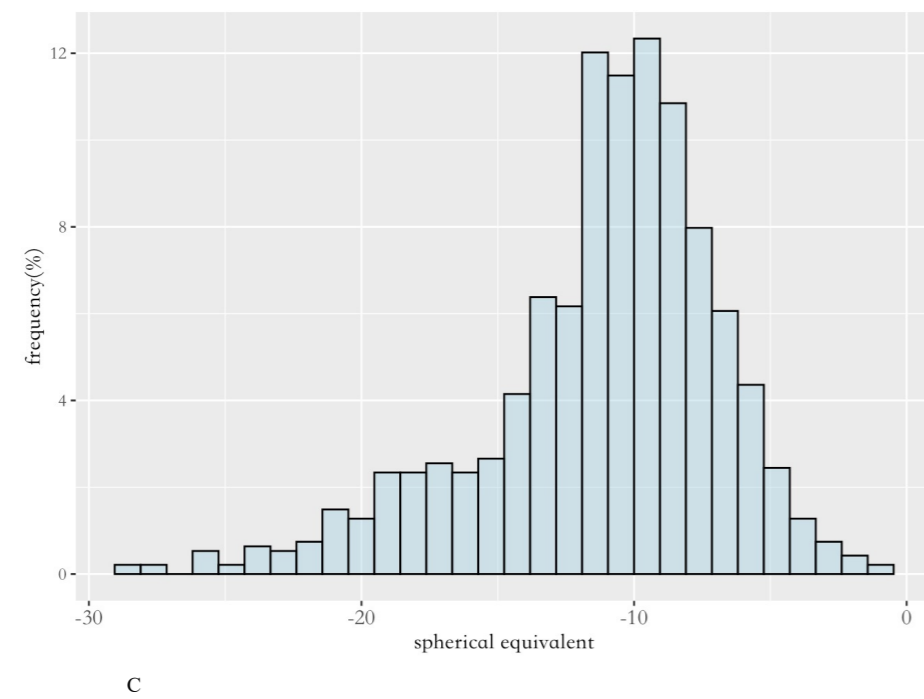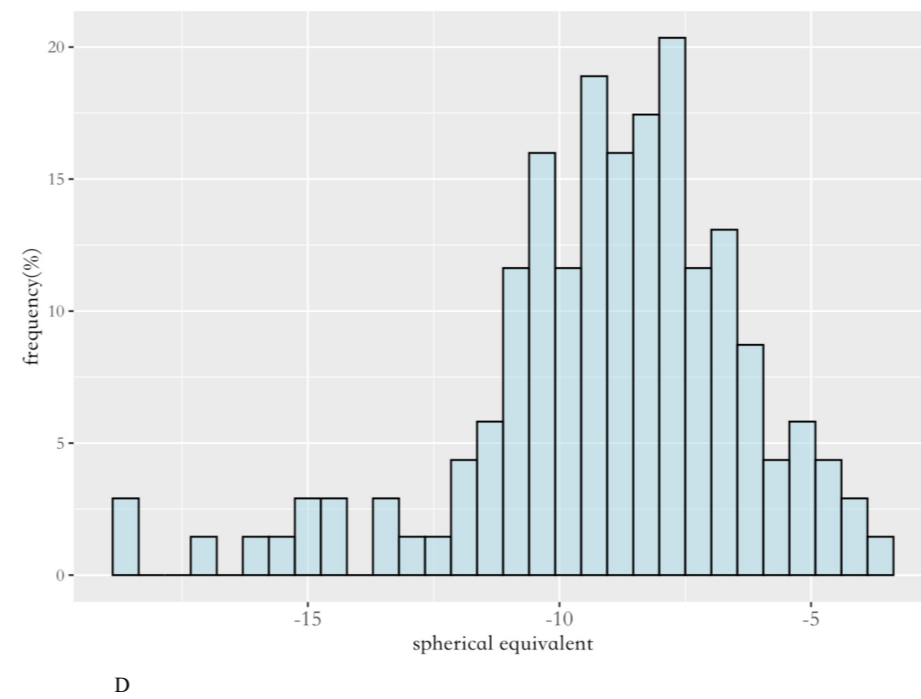

Supplement: Supplementary Figure 2 — Distribution of MAE of predicted spherical equivalent. (A) ResNet-50; (B) Inception-v3; (C) Inception-ResNet-v2. [file Image_2.pdf]

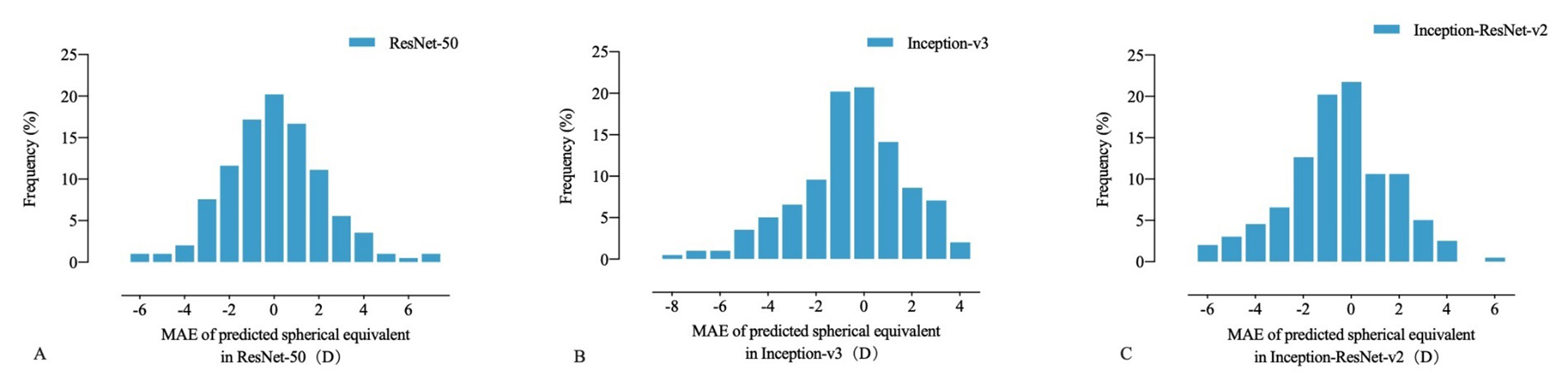

Supplement: Supplementary Figure 3 — Distribution of MAE of predicted spherical equivalent in the external validation set. (A) ResNet-50; (B) Inception-v3; (C) Inception-ResNet-v2. [file Image_3.jpg]
