# Supplementary material for: Prediction of Refractive Error Based on Ultrawide Field Images With Deep Learning Models in Myopia Patients
Source: Front Med (Lausanne). 2022 Mar 30;9:834281. doi: 10.3389/fmed.2022.834281 (PMC9007166; doi:10.3389/fmed.2022.834281)
Supplement: Supplementary Table 1 — Patient characteristics of the training set, test set, whole data set and the external validation set. [file Table_1.DOCX]

|  | **Training Set** | **Test Set** | **Total** | **External Validation** | **P value** |
| --- | --- | --- | --- | --- | --- |
| No. | 790 | 197 | 987 | 133 |  |
| Age (year)† | 28.14$\pm$7.14 | 28.56$\pm$6.62 | 28.23$\pm$7.04 | 27.76$\pm5$.53 | 0.419 |
| Gender (Female/Male)§ | 559/231 | 154/43 | 713/274 | 110/23 | <0.01 |
| Spherical Equivalent (D)† | -11.46$\pm$4.61 | -10.02$\pm$3.34 | -11.17$\pm$4.41 | -9.03$\pm$2.79 | <0.01 |
| Axial Length (mm)† | 27.95$\pm$2.14 | 27.40$\pm$1.63 | 27.85±1.99 | 27.19$\pm1.50$ | <0.01 |
| Intraocular Pressure(mmHg) | 15.47$\pm$2.81 | 15.08$\pm$2.52 | 15.39$\pm$2.76 | NA |  |
| K1† | 42.80$\pm$1.50 | 42.95$\pm$1.52 | 42.83$\pm$1.51 | 42.70$\pm$1.19 | 0.189 |
| K2† | 44.43$\pm$1.65 | 44.50$\pm$1.72 | 44.44$\pm$1.66 | 44.15$\pm$1.35 | 0.147 |

Supplemental Table 1. The characteristic of the participants

† Kruskal-Wallis rank sum test, §chi-squared test
